# Supplementary material for: Hybrid Models and Biological Model Reduction with PyDSTool
Source: PLoS Comput Biol. 2012 Aug 9;8(8):e1002628. doi: 10.1371/journal.pcbi.1002628 (PMC3415397; doi:10.1371/journal.pcbi.1002628)
Supplement: Text S4 — Complete source code for the PyDSTool package (version 0.88.120504). Includes API documentation and help files linking to web pages. This file is identical to the current public release on Sourceforge.net. (ZIP) [file pcbi.1002628.s004.zip › PyDSTool/html/PyDSTool.Toolbox.ParamEst.LMpest-class.html]

xml version="1.0" encoding="ascii"?


PyDSTool.Toolbox.ParamEst.LMpest


| Home | Trees | Indices | Help | PyDSTool |
| --- | --- | --- | --- | --- |

Package PyDSTool ::
Package Toolbox ::
Module ParamEst ::
Class LMpest

[hide private]

[frames] | no frames]

# Class LMpest

source code

```
    object --+        
             |        
common.Utility --+    
                 |    
          ParamEst --+
                     |
                    LMpest
```

---

Unconstrained least-squares parameter and initial condition optimizer
for n-dimensional DS trajectories. Fits N-dimensional parameter
spaces.

Uses MINPACK Levenberg-Marquardt algorithm wrapper from
SciPy.minimize.


|  |  |
| --- | --- |
| Instance Methods | [hide private] |

|  |  |
| --- | --- |
| setAlgParams(self, changed\_parDict=None)  Set algorithmic parameters. | source code |

|  |  |
| --- | --- |
| run(self, parDict=None, extra\_pars=None, verbose=False)  Begin parameter estimation run. | source code |

|  |  |
| --- | --- |
| \_make\_res\_float(self, pars)  Returns a function that converts residual vector to its norm (a single floating point total residual). | source code |

|  |  |
| --- | --- |
| gradient\_total\_residual(self, x, eps=None, pars=None, use\_ridder=False)  Compute gradient of total residual (norm of the residual function) at x as a function of parameter names specified (defaults to all free parameters). | source code |

|  |  |
| --- | --- |
| Hessian\_total\_residual(self, x, eps\_inner=None, eps\_outer=None, pars=None, use\_ridder\_inner=False, use\_ridder\_outer=False)  Compute Hessian of total residual (norm of the residual function) at x as a function of parameter names specified (defaults to all free parameters), USING FINITE DIFFERENCES. | source code |

**Inherited from `ParamEst`**:
`__init__`,
`evaluate`,
`find_logs`,
`iterate`,
`key_logged_residual`,
`par_sensitivity`,
`pars_array_to_dict`,
`pars_dict_to_array`,
`pars_to_ixs`,
`resetParArgs`,
`reset_log`,
`setFn`,
`show_log_record`,
`weighted_par_sensitivity`

**Inherited from `object`**:
`__delattr__`,
`__getattribute__`,
`__hash__`,
`__new__`,
`__reduce__`,
`__reduce_ex__`,
`__repr__`,
`__setattr__`,
`__str__`


|  |  |
| --- | --- |
| Properties | [hide private] |

**Inherited from `object`**:
`__class__`


|  |  |
| --- | --- |
| Method Details | [hide private] |

|  |  |
| --- | --- |
| setAlgParams(self, changed\_parDict=None) | source code |

Set algorithmic parameters.

Overrides: ParamEst.setAlgParams: *(inherited documentation)*

|  |  |
| --- | --- |
| run(self, parDict=None, extra\_pars=None, verbose=False) | source code |

Begin parameter estimation run.

parDict can include arbitrary additional runtime arguments to the
residual function.

If tmesh is not supplied an attempt will be made to create one from
the goal trajectory's independent domain limits, if the trajectory has
been provided. Default mesh resolution is 20 points.

Overrides: ParamEst.run

|  |  |
| --- | --- |
| \_make\_res\_float(self, pars) | source code |

Returns a function that converts residual vector to its norm (a single
floating point total residual).

(Helper method for gradient and Hessian)

|  |  |
| --- | --- |
| Hessian\_total\_residual(self, x, eps\_inner=None, eps\_outer=None, pars=None, use\_ridder\_inner=False, use\_ridder\_outer=False) | source code |

Compute Hessian of total residual (norm of the residual function) at x
as a function of parameter names specified (defaults to all free
parameters), USING FINITE DIFFERENCES.

Option to use different eps scalings for the inner gradient
calculations versus the outer gradient of those values.

It might be more accurate to calculate the Hessian using a QR
decomposition of the Jacobian.

  


| Home | Trees | Indices | Help | PyDSTool |
| --- | --- | --- | --- | --- |

|  |  |
| --- | --- |
| Generated by Epydoc 3.0.1 on Fri May 4 15:24:08 2012 | http://epydoc.sourceforge.net |
